# Towards ethical guidelines for dealing with unsolicited patient emails and giving teleadvice in the absence of a pre-existing patient-physician relationship — systematic review and expert survey

**DOI:** 10.2196/jmir.2.1.e1

**Published:** 2000-02-24

**Authors:** Gunther Eysenbach

**Keywords:** Internet, Ethics, Quality of Health Care, Referral and Consultation, Remote Consultation, Physician-Patient Relations, Professional-Patient Relations, Medical History Taking, Teleadvice, Electronic Mail, Chat, Newsgroup

## Abstract

**Background:**

Many health information providers on the Internet and doctors with email accounts are confronted with the phenomenon of receiving unsolicited emails from patients asking for medical advice. Also, a growing number of websites offer "ask-the-doctor" services, where patients can ask questions to health professionals via email or other means of telecommunication. It is unclear whether these types of interactions constitute medical practice, and whether physicians have the ethical obligation to respond to unsolicited patient emails.

**Objective:**

To improve the quality of online communication between patients and health professionals (physicians, experts) in the absence of a pre-existing patient-physician relationship or face-to-face communication, by preparing a set of guiding ethical principles applicable to this kind of interaction.

**Methods:**

Systematic review of the literature, professional, and ethical codes; and consultation with experts.

**Results:**

Two different types of patient-physician encounters have to be distinguished. "Traditional" clinical encounters or telemedicine applications are called "Type B" interactions here (Bona fide relationship). In comparison, online interactions lack many of the characteristics of bona fide interactions; most notably there is no pre-existing relationship and the information available to the physician is limited if, for example, a physician responds to the email of a patient who he has never seen before. I call these "Type A" consultations (Absence of pre-existing patient-physician relationship). While guidelines for Type B interactions on the Internet exist (Kane, 1998), this is not the case for Type A interactions. The following principles are suggested: Physicians responding to patients' requests on the Internet should act within the limitations of telecommunication services and keep the global nature of the Internet in mind; not every aspect of medicine requires face-to-face communication; requests for help, including unsolicited patient questions, should not be ignored, but dealt with in some appropriate manner; informed consent requires fair and honest labeling; health professionals and information providers must maintain confidentiality; health professionals should define internal procedures and perform quality control measures.

**Conclusions:**

Different media are appropriate at each point on the continuum between dispensing general health information and handling patient problems that would require the practice of medicine to solve. For example, email is a sufficiently capable medium for giving out general health information, while diagnosis and treatment usually requires at least advanced telemedical technology. Patients have to be educated that it is unethical to diagnose and treat over the Internet in the absence of a pre-existing patient-physician relationship, and if the interaction is limited to a single email. More research is needed to establish more evidence regarding situations in which teleadvice is beneficial and efficient.

*The green paper set forth below concerns ways to improve the quality of online communication between patients and health professionals (physicians, experts) in the absence of a pre-existing patient-physician relationship or face-to-face communication, and takes a first step towards proposing a set of ethical standards for this kind of interaction. These principles are preliminary, and were drawn up as a result of a systematic review of the literature; consultation with professional organizations, associations and bodies; and a workshop at the AMIA Fall Conference in 1998; and with input by an international expert panel of the Internet Healthcare Coalition (IHC), Society of Internet in Medicine (SIM) and the American Medical Informatics Association (AMIA). In order to provide input into a more general set of guidelines, the Code of e-Health Ethics currently being set up by the e-Health community, this paper was also circulated among the participants of the e-Health Ethics Summit, organized by the Internet Healthcare Coalition in Washington D.C., January 31 - February 2, 2000. Aspects of this paper were subsequently included in the "provision of medical practice on the Internet" section of the Code.*

*The author will accept comments on the paper starting today until March 31, 2000. Comments may be sent via electronic mail to ey@yi.com. All comments received will be considered in the context of issuing a final white paper, and if the comments are substantial, the author of the comment will become a co-author of the final paper. The green paper has been published in the Journal of Medical Internet Research solely as a means to facilitate the public's access to this document, and to provide an additional means of notifying the public of the solicitation of public comment on the proposed White Paper, which is scheduled to be submitted for publication in April 2000.*

## Introduction

While telemedicine services and physician telephone services have been around for several decades, the unprecedented popularity of the Internet has greatly facilitated patients' access to physicians and led to a new form of communication between patient and health care professional - "text-only" communication in emails and other venues, in the absence of a pre-existing relationship (in this paper called "Type A" communication). Every physician who has published his email address or who runs a medical website receives unsolicited emails from patients he or she has never seen before. Patients use email to ask medical questions to physicians unknown to them, or sometimes even describe their symptoms and expect a remote diagnosis. Health portal sites and specialized services responded to this consumer demand for "virtual interaction" with physicians, and have set up "ask-the-expert" services and "cyberdoctor" services, which offer such advice for free or for a charge.

The intent of this paper is to prepare a consensus on a set of guidelines for health professionals on dealing with unsolicited patient emails, and for physicians and nurses working for medical "ask-the-doc" or "ask-the-expert" services on the Internet.

### Terminology and definition of the issues: Type A and Type B interactions

The digital revolution and the Internet have opened new ways for health providers and consumers to interact. Aside from the fact that the Internet allows transmission of high-level, high-bandwidth telemedicine applications, it also allows simple exchange of electronic, written messages between patient and health professional, which can be seen as a form of "low-level," "low-tech," "low-cost" telemedicine. Other terms used for this kind of interaction are "teleconsultation" or "teleadvice."

**Table 1:** Differences in Type A and Type B relations

	**Type A encounters** (online interaction between patient and health professional in the **a**bsence of pre-existing relationship)	**Type B encounters** (**b**ona fide relationship; encompasses traditional clinical encounters and telemedicine)
Contractual relationship before the encounter	No pre-existing relationship	Mostly pre-existing contractual relationship
Responsibility	Physician has not taken explicit responsibility for the patient	Physician has taken explicit responsibility for the patient
Contact initiation	Contact usually initiated by the patient	Contact scheduled or initiated by physician, or by patient
Transmission of information	Usually only text (e-mail consultations and chats)	Face-to-face, sound, video, images
Access to information	Information limited to what the patient provides	Physician has access to health record or other channels to obtain more information
Patient's knowledge of the physician	Patient doesn't know the physician in advance	Patient usually knows the physician or has a referral
Physician's knowledge of the patient	Physician doesn't know the patient	Physician usually knows the patient
Physician's preparation to receive requests from patients	Physician is not prepared to get requests from patients*	Physician explicitly offers advice

^*^ Does not apply to "ask-the-expert" services

Different technologies may be used for teleadvice:

- electronic mail (email), allowing "private" exchange of messages
- newsgroups, allowing "public" discussions
- chatrooms, allowing direct written communication via keyboard

All these venues for "cybermedicine" may cater to patient-physician interactions that are fundamentally different from classical telemedicine applications in a number of ways (see Table 1).

In "traditional" clinical encounters or telemedicine applications, there is either a pre-existing patient-physician relationship or, if the patient comes to the practice for the first time, the professional at least has access to the patient's electronic health record, or can consult with the referring physician. For the purpose of this paper, I call these traditional interactions "Type B" interactions (**B**ona fide relationship).

On the contrary, online interactions lack many of the characteristics of bona fide interactions; most notably there is no pre-existing relationship. I call these "Type A" consultations (**A**bsence of pre-existing patient-physician relationship). Note that Tom Ferguson calls the latter Type I and the former Type II interactions, "Type I because this developed first" [Ferguson T, personal communication].

### Subgroups of Type A encounters

Examples for (and subgroups of) these "atypical" telemedicine encounters are:

- a patient sending an "unsolicited" email to a physician (A1)
- "ask-the-expert" services on the Internet, where consumers are invited to ask medical questions which are forwarded to medical experts (A2)
- a patient soliciting help from a physician by posting a public request for help on a newsgroup or website, to which a physician replies (A3)

**Figure 1:**
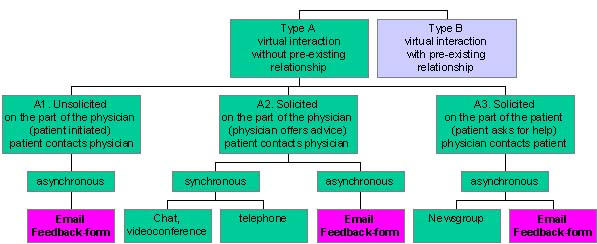
Subgroups of Type A interactions

These interactions differ from each other because the physician has taken different levels of action (and perhaps responsibility) - in the first case, unsolicited patient email (A1), only the patient has taken action; in the second case, (A2), the physician is part of a team that volunteered and explicitly offered to answer patient questions; and in the third case, (A3), the physician replies to a patient request (see Figure 1).

In all of these cases, the relationship between patient and physician is less well-defined and more prone to misunderstandings than in traditional Type B patient-physician encounters. For example, in Type B encounters the patient is used to receiving a reply, which may not be the case in Type A encounters. Similarly, the situation is difficult for the physician, as he or she may not be sure about the ethical duties and the legal consequences of his or her actions. Guidelines may help to better define such contacts to avoid misunderstandings [1,2]. There have also been concerns that what we call Type A encounters here may "disturb delicate balances in the patient-physician relationship, widen social disparities in health outcomes, and create barriers to access" [3]. In the context of newsgroup-like interactive communication and information listservers, where patients can ask questions to experts, Spielberg criticized that such a system "bypasses existing patient-physician relationships, since it does not facilitate communication within them" [4].

### What is different on the Internet?

It should be noted that Type A consultations may also occur outside of cyberspace, in the form of patients calling or writing letters to physicians. However, in comparison with these interactions, there are differences in Internet-enabled consultations; for example:

- communication is nearly anonymous, thus more impersonal
- communication is more informal
- communication is global
- access to physicians on the Internet is easier than in the real world

**Figure 2:**
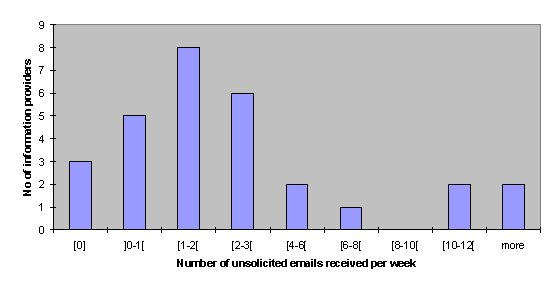
Number of unsolicited emails received per week, according to a survey of 23 health information providers

All of these factors, especially that the Internet allows near-anonymous communication and lowers the barrier for consumers to interact with providers, contribute to the fact that the demand for Type A interactions has reached an unprecedented level in the history of medicine. Every doctor or medical information provider who runs a medical website which provides his or her email address will sooner or later be faced with the problem of getting unsolicited emails from patients asking him or her for medical information or advice. In a survey of 23 Internet health information providers (mostly doctors also acting as webmasters), participants were asked, "How many unsolicited patient emails do you get per week?" The numbers given ranged between 0 and 50 emails, with a mean value of 4.4 (STD 9.47) and a median of 1 email per week (see Figure 2). In the same survey, 62% of the information providers said that "unsolicited emails from patients represents a significant unresolved problem on the Internet" [1].

There are further differences between Type A email-teleadvice compared to Type B telemedicine, which primarily concern privacy and security concerns inherent to email and other insecure venues on the Internet. Aspects of these concerns have been explored elsewhere [5].

### Existing guidelines

The most important guideline in the context of "virtual" patient-physician interactions on the Internet is the guideline published by the AMIA Internet Working Group, which however explicitly focuses on "computer-based communication between clinicians and patients within a contractual relationship in which the health care provider has taken on an explicit measure of responsibility for the client's care" (emphasis added by the author) [5], thus applying only to Type B interactions. Although many of the principles of email communication in the clinical context also apply to email communication in Type A interactions, the more ill-defined Type A interactions between providers and consumers in which no contractual relationship exists require additional exploration. It was not before several papers published in the Journal of the American Medical Association (JAMA) [5-8] drew attention to the fact that these kinds of interactions exist that the need to develop guidelines for Type A interactions was acknowledged. In the same issue, Spielberg pointed out that "email communications are not merely virtual approximations of medical practice, they are very real exchanges of information, advice and emotions. (...) Electronic communication, as a novel technology, is neither inherently unethical nor readily acceptable for medical practice. Rather, the emergence of electronic communication launches a reexamination of the necessary values for good communication in the patient-physician relationship" [8].

### Aim of this paper

This paper tries to analyze and synthesize issues arising from Type A interactions and intends to summarize existing evidence, opinions, and ethical codes relevant to the issues. A set of principles will also be proposed.

## Methods

### Database Searches

I tried to primarily identify publications describing empirical data or legal and ethical standards on patient-physician interaction on the Internet (in email, chat, and newsgroups). MEDLINE was searched from 1966-1999 with the PubMed search strategy:


(("remote consultation"[majr] OR "Referral and Consultation"[majr] OR 
"Physician-Patient Relations" OR "Professional-Patient Relations" OR "teleadvice" OR 
"advice" OR "unsolicited email*") AND (internet OR "email*" OR "e-mail*" OR 
"electronic mail*" OR "chat" OR "newsgroup*" OR "usenet')


A total of 200 publications were found and screened on the basis of the abstract and the title. Most of these publications dealt with physician-to-physician telemedicine applications or physician-patient interactions in the framework of a pre-existing patient-physician relationship and were therefore only of marginal interest for this review.

### Review of ethical and professional codes

The professional and ethical codes of the following organizations (countries) were reviewed: American Medical Association (USA); Bundesärztekammer and Landesärztekammern (Germany); Ethical guidelines for telemedicine adopted by the Standing Committee of European Doctors; Swiss Medical Federation FMH (Switzerland); General Medical Council (UK); World Medical Association (WMA).

### Contacts to experts

A wide range of experts was consulted to elucidate the issues. A workshop entitled "Unsolicited emails from patients to health information providers and doctors on the WWW asking for medical advice - how to handle them?" was organized by the author at the AMIA Fall Symposium, Orlando(Florida), Nov 7, 1998. Letters were written to legal experts at professional medical bodies. Requests for comments were posted on various mailing lists and a panel of experts (listed under Acknowledgements) identified themselves and provided input.

## Results

### Available Evidence

#### What are patients asking online?

Three studies exist in the medical literature that have analyzed the nature and content of patient requests:

- Widman & Tong [9] analyzed 70 unsolicited emails sent by patients over a period of 12 months. The inquiries (mostly concerning cardiac arrhythmias, as they were sent in response to a website focusing on this topic) were questions about diagnosis (15), therapy (48), prognosis (1), and patient education (6).
- Eysenbach & Diepgen [2] provided a more thorough analysis of 209 emails sent to a university department of dermatology in a four-month period between April and August 1997. Forty percent of all emails could have been answered by a librarian, 28% of all emails were suitable to be answered by a physician via email alone, and in 27% of the cases any kind of consultation would not have been possible without seeing the patient. In 34% of the cases, patients wanted to have general information about a condition, and three-quarters of the messages (75%) contained 1 or more specific questions, mostly about therapy (30%) or requests for information about a "specialist" to treat a given condition (15%). Eleven percent gave a list of symptoms and wanted to hear a diagnosis.
- Borowitz & Wyatt [7] analyzed 1,001 requests from patients sent between November 1995 and June 1998 to the Division of Pediatric Gastroenterology at a children's medical center. Contrary to the previous two studies, these appear to be solicited requests. In 69% of the requests, there was a specific question about the cause of a particular child's symptoms, diagnostic tests, and/or therapeutic interventions. In 112 of the requests (9%), the requester sought a second opinion about diagnosis or treatment for a particular child, and 272 consultations (22%) requested general information concerning a disorder, treatment, or medication without reference to a particular child.

In summary, it can be learned from these studies that the spectrum of questions ranges from very general questions to questions that would involve medical decision-making. The latter constitute about 27% in one study [2]. It can also be learned that patients are not always able to distinguish between questions that are suitable to be answered via email and those that aren't.

#### What are physicians doing on the Internet?

Very little is known about what physicians are actually doing on the Internet and to what degree this constitutes medical practice.

- Culver and colleagues [10] analyzed 1,658 consecutive messages on a particular online discussion group during a 5-month period. Of all messages, 55.9% (927) addressed a medical topic. Of these, 79% (732) provided medical information, of which 5.1% (37) were authored by trained health professionals. Personal experience was the basis of information provided in 13.5% of the professionals' messages, while no source was given as the basis of information provided in 29.8% of the nonprofessionals' messages and 67.6% of the professionals' messages. A published source was cited in 9.2% of the nonprofessionals' and 18.9% of the professionals' messages.
- Eysenbach and Diepgen [1] sent an unsolicited email in December 1997 and January 1998 from a fictitious patient describing an acute dermatological problem to 58 physicians and webmasters to explore the response rate and the types of responses in terms of amount of information given. Fifty percent responded to the fictitious patient request; of those who responded, 31% refused to give advice without having seen the lesion, 59% explicitly mentioned the correct "diagnosis" in their reply, and 17% gave detailed treatment advice. Ninety-three percent recommended that the patient see a physician. Two different arguments were brought forward in the replies: the impossibility of making a diagnosis via email without an examination ("The diagnosis is unclear because we cannot look at your exanthema."), and/or lack of resources and/or mandate to "reply to all the enquiries of this kind that we receive." Some of these responses were probably standard replies.
- A similar email from a fictitious patient was also sent to commercial "cyberdocs" who explicitly offered medical advice on the Internet [11]. Ten free and 7 charging cyberdocs were contacted. Ten cyberdocs responded. Three declined to give advice because dermatology was not their area of expertise. Seven cyberdocs provided advice (2 for free, 5 for a charge). The advice given by 5 cyberdocs was accurate, and the "correct" diagnosis herpes zoster was mentioned. In the remaining 2 cases the advice was highly questionable: one cyberdoc recommended a homeopathic medicine, the other unusual methods such as drinking rain water and eating red clover and dandelion.
- Sandvik [12] repeated these studies by sending an email from a fictitious incontinent woman to 75 websites providing information on this topic. Sixty-six percent of the sites responded to the email request for advice. Messages were also sent to two newsgroups, but the results are not reported.

In summary, it can be stated that a significant number of physicians on the Internet do not confine their interactions with patients to giving general advice, but also make diagnoses and give therapeutic hints.

#### What constitutes medical practice - and can a patient-physician relationship be established online?

Although the a large part of the daily practice of medicine encompasses giving health information, providing emotional support, and coordinating care, legislation defines "practicing medicine" much more narrowly, by focusing on diagnosis and treatment.

For example, the US State of New Hampshire defines the practice of medicine as follows:

Any person shall be regarded as practicing medicine under the meaning of this chapter who shall diagnose, operate on, treat, perform surgery, or prescribe for or otherwise treat any disease or human ailment, whether physical or mental. "Surgery" means any procedure, including but not limited to laser, in which human tissue is cut, shaped, burned, vaporized, or otherwise structurally altered, except that this section shall not apply to any person to whom authority is given by any other statute to perform acts which might otherwise be deemed the practice of medicine. "Laser" means light amplification by stimulated emission of radiation.

[State of New Hampshire, RSA 329:1, amended June 18, 1997. Available from: http://www.state.nh.us/gencourt/bills/chaptered/97chapters/0214-hb0718.htm]

**Figure 3:**
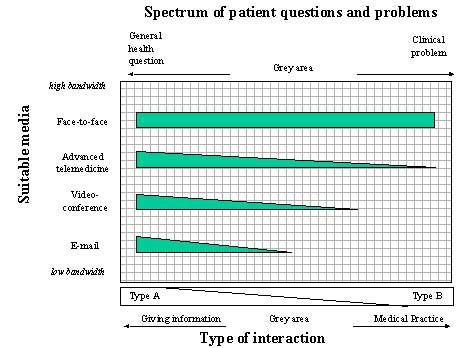
Different media are appropriate at each point on the continuum between dispensing general health information and handling patients' problems which would require the practice of medicine to solve. For example, email is a sufficiently capable medium for giving out general health information, while diagnosis and treatment usually requires at least advanced telemedical technology. Likewise, when dispensing general health information, a Type A relationship between the patient and the physician is sufficient; for practicing medicine, a Type B relationship is desirable. The difficulty is that there is no clear-cut line between the two extremes - and it is in this grey area that the majority of patient-physician interactions on the Internet take place

That lawyers focus on the concepts of diagnosis and treatment has its justification, as many other people also give health information, and provide emotional support or health advice, without being physicians and having a medical license; for example, journalists, webmasters of health websites, librarians, priests, or hair stylists. While there seems to be consensus that giving general health information is not "practicing medicine," and that the process of diagnosing and treating clearly constitutes practicing medicine, there is a large grey area between these two extremes (see Figure 3).

A significant part of patient-physician interactions on the Internet takes place in this grey area. Part of the problem is that "treatment" is another ill-defined concept - do we "treat" someone, if we give him or her advice related to his orher health? Physicians would say yes, as many medical conditions can in fact be treated simply by giving advice. Journalists would perhaps say no, as otherwise they would practice medicine if they publish health stories. What is the difference between health advice given by a physician and health advice given by a journalist? One difference is that the former is usually given face-to-face, while the latter is given via a medium. In the age of telemedicine, however, face-to-face interaction cannot be a suitable criterion to define medical practice - especially not on the Internet, where everything we do is through a medium. Another difference is that the physician listens to our problem and then gives tailored advice hoping that the patient will act upon it, while the journalist only listens to the "collective voice" of his target group and gives more general advice without knowing who acts on this information. Thus, the feedback loop of listening to an individual and reacting specifically to his needs could be a guiding principle to define medical practice: The more health information is personalized and tailored to the individual, and the more it encourages the receiver to act upon the advice, the more we are moving within the continuum from giving general health advice towards attempting to treat, and therefore practicing medicine. This would also imply that expert systems and dynamic web pages providing tailored information on the basis of feedback forms filled in by users may well be considered as practicing medicine. Still, there remains a huge grey area. For example, telephone advice services such as the British NHSDirect, where health professionals advise patients whether their condition justify a doctor visit, certainly provide personalized information on which the receiver acts directly, without being necessarily considered as practicing medicine. Thus, different standards for different kinds of advice given may apply. Much of the confusion, controversy and debate about the legitimacy of giving medical teleadvice has also to do with the fact that different people have different thoughts on what exactly is meant by teleadvice; for example, giving general health information, giving personalized health information, or even diagnosis or treat.

On many medical websites, particularly on "ask-the-expert" services, health information providers publish disclaimers which aim to reduce the risk of misunderstandings on the nature of such advice. It has been noted that the legal ramifications of such disclaimers are unclear: "Statements claiming that medical advice or second opinions rendered via the Internet do not constitute the practice of medicine have yet to be tested for legal effect, though such disclaimers rarely insulate practitioners from the prevailing standards of care" [8].

Thus, disclaimers may well help patients to become aware on the limitations of telecommunication services, but they are unlikely to liberate physicians from liability claims.

In a letter to JAMA, R. Neill pointed to the fact that: "In the United States, a patient-physician relationship is established when a physician exercises independent medical judgment on the patient's behalf, whether explicitly or implicitly. One legal test of the relationship is embodied in the question of reliance: did the patient reasonably rely on the physician's judgment [13]? Keeping in mind these precepts, physicians clearly have the capacity to establish patient-physician relationships using e-mail" [14].

Not all advice can be treated equally, as there is a spectrum of patient questions and physician replies (as shown above) that ranges from "general information" to "clinical issues." It is necessary to make a distinction between such general responses and clinical advice. M. Howard mentioned in another article that: "A physician offering advice by email will be liable for unfavorable results of that advice if a reasonable person would have understood the physician to be offering therapy. A general response to a vague question will probably not be sufficient to establish a physician-patient relationship with a person not already a patient of the practice" [15].

Thus, there seems to be consensus that physicians can indeed establish a patient-physician relationship online, and that it depends on their reply and their actions whether the interaction can be considered to be an act of medical practice or just an act of "information brokerage." However, there will always be a grey area, and it is the responsibility of the physician to act according to where on the continuum (Figure 3) the patient's problem is located, and according to which "media" of interaction are available. Moreover, it is essential to clearly state the nature of the interaction to the patient.

#### Ethical and professional codes

A number of ethical and professional codes were reviewed concerning giving advice by telecommunication.

##### World Medical Association

The World Medical Association (WMA) is currently consulting its National Medical Association members around the world with a view to drawing up new ethical guidelines on telemedicine.

##### Standing Committee of European Doctors

Ethical guidelines for telemedicine adopted by the Standing Committee of European Doctors demand that: "Where a direct telemedicine consultation is sought by the patient, it should normally only take place when the doctor has an existing professional relationship with the patient, or has adequate knowledge of the presenting problem. (...) Preferably, all patients seeking medical advice should see a doctor in a face to face consultation, and telemedicine should be restricted to situations in which a doctor can not be physically present within acceptable time" [16].

Both are rarely the case on the Internet in Type A interactions - neither is there, by definition, an existing professional relationship, nor are remoteness or physical disabilities the main reasons for consulting Internet doctors [2].

##### Germany

German physicians who give individual advice to patients by mail or email would clearly violate their professional code, which explicitly provides that:

...no physician may give individual medical treatment, including medical advice, neither exclusively by mail . . . nor exclusively over communication media or computer communication networks.

(B.II. §7, Par. 3; German Model Regulations for the Professional Code)

A spokesperson from a German physician association recently insisted that "any medical advice must be given face-to-face. This has been always like this, and it will remain like this" [17].

##### Switzerland

In Switzerland the professional code [18] says:

The regular treatment solely on the basis of written, by phone or electronically transmitted information or reports from third parties is incompatible with a genuine conduct of the profession.

(Standesordnung FMH, 12 Dec 1996; Article 7)

Note that the word "regular" is used, which does not exclude an initial or occasional contact. Recognizing that advice via telecommunication is part of medical reality, and that there is a demand on the patients' side for Internet teleadvice services, Swiss legal experts have taken steps to define a framework for teleadvice services. A main requirement for such services is that they define their offer thoroughly in terms of:

- Defining of reply times
- Defining whether each question will be answered, or whether there is a selection
- Defining whether only requests from Swiss citizens are answered, or also international requests
- A disclaimer saying that not all questions can be answered by email, and that the patients may have to see a doctor

If there is no pre-existing patient-physician relationship, physicians can charge patients only on a private basis; coverage by the social security health insurance is not possible.

##### HON-Code

The Swiss Health On the Net Foundation (HON) does not, in its HON Code of Conduct, provide any specific principles for giving advice via email; but referring to information on medical websites, it is stated as one of the principles that: "Information should be designed to support, not replace, the relationship that exists between a patient/site visitor and his/her existing physician" [19].

##### United States

The Ethics Committee of the American Medical Association (AMA) has drawn up recommendations for "Physician Advisory or Referral Services by Telecommunications" [20]. These acknowledge that teleadvice services can be useful for the public and are, compared to the other professional codes listed above, much more clear as to what can be considered reasonable and what is ethically critical (diagnosis and especially therapy). Also relevant is the policy statement on phone counseling (which could also be applied to "ask-the-expert" services on the Internet) and - in terms of quality management of such services - the policy on "disease management and demand management" (all given below).

**AMA Current Opinions of the Council on Ethical and Judicial Affairs**

***E-5.025 Physician Advisory or Referral Services by Telecommunications***

Telecommunication advisory services, by way of phone, fax, or computer, distinct from an existing physician-patient relationship can be a helpful source of medical information for the public. Often, people are not sure where to turn for information of a general medical nature or do not have easy access to other sources of information. Individuals also may be embarrassed about directly bringing up certain questions with their physicians. Although telecommunication advisory services can only provide limited medical services, they can be a useful complement to more comprehensive services, if used properly.

Any telecommunication advisory service should employ certain safeguards to prevent misuse. For example, the physician responding to the call should not make a clinical diagnosis. Diagnosis by telecommunication is done without the benefit of a physician examination or even a face-to-face meeting with the caller. Critical medical data may be unavailable to the physician. Physicians who respond to callers should therefore act within the limitations of telecommunication services and ensure that callers understand the limitations of the services. Under no circumstances should medications be prescribed.

Physicians who respond to the calls should elicit all necessary information from the callers. When callers are charged by the minute, they may try to hurry their calls to limit their costs. As a result, important information may not be disclosed to the physician. Physicians should also ensure that callers do not incur large bills inadvertently or without understanding the billing system.

Physician referral services can also offer important information to the public. Referral services are often provided by medical societies, hospitals and for-profit entities. To ensure that the service bases its recommendation on medically legitimate considerations rather than the likelihood of being paid by the physician, when the service charges physicians a fee to participate, physicians should not pay the service per referral. Also, callers should be told how the list is created. For example, callers should be informed whether the list includes physicians who pay a flat fee to be listed, members of a particular hospital staff or medical society, or physicians who meet some general quality-based criteria.

While these safeguards are described as applying primarily to telephone services, they should be considered equally applicable to any other communication media, such as radio, or television, in which the physician and patient do not meet face-to-face. Issued June 1994; Updated June 1996. (I, IV, VI)

**Policies of the AMA House of Delegates**

***H-160.935 Policy on Phone Counseling***

The AMA recommends the following statements on phone counseling: (1) Medical phone counseling services must appoint a physician director. Such services are not absolved of that responsibility by a disclaimer to the callers. A physician director must be ultimately responsible for the telephone triaging of patients in a given system. (2) A physician director must be responsible for: (a) Providing and updating protocols and algorithms for phone counseling by non-physicians. (b) Identifying high-risk patients who must be directly and immediately referred to physicians at all times. (c) Supervision and review of second-level triage provided by advanced nurse practitioners and physician assistants. (d) Ensuring permanent records of all calls received. (e) Maintaining accountability for the patient until a referral has been effected with an accepting physician. (3) Urges quality assurance programs be developed by national accrediting agencies that address issues raised by phone counseling centers. (BOT Rep. 2, A-97)

***H-285.944 Disease Management And Demand Management***

.... phone counseling and triage centers should routinely compile outcome information on all calls handled, and should modify their operating policies and referral protocols as needed to enhance the effectiveness of the service.

(14) Telephone triage centers should routinely inform primary or principal care physicians of the disposition of all calls received from their patients.

(15) Telephone counseling and triage should be performed by health professionals with a level of knowledge and training no less than that of a registered nurse.

(16) Qualified physicians should be readily accessible for consultation and second-level triage to the nurses or other health professionals providing telephone counseling or triage.

(17) Physicians performing second level triage for telephone triage centers should be compensated for such services by the center or sponsoring health plan.

(18) Compensation for individuals performing telephone counseling and triage should not be based on the number or the disposition of calls handled.

(19) Organizations that provide telephone triage services should provide such services 24 hours a day on a year-round basis and calls should be handled as expeditiously as possible. (CMS Rep. 3, I-97; Reaffirmed by Sub. Res. 707, A-98)

##### Great Britain

Following reports in the literature on "cyberdoctors" [11], the General Medical Council (GMC) has drafted a note on "Providing advice and medical service on-line or by telephone", which is reprinted below. It is noteworthy that it does not strictly preclude any email advice (such as in the German professional code), but leaves the responsibility and the decision to the judgment of the individual physician.

**GMC-General Medical Council (UK) "Good Medical Practice"**

***Providing advice and medical services on-line or by telephone (November 1998)***

Giving advice by telephone is part of many doctors' day-to-day relationship with their patients. In some circumstances providing advice by telephone or computer link may be essential, for example, where patients are geographically isolated from their doctor.

However the use of phone or e-mail should not diminish the quality of care patients receive. Consultations and prescribing by phone or e-mail may seriously compromise standards of care where:

- The patient is not previously known to the doctor, and
- No examination can be provided, and
- There is little or no provision for appropriate monitoring of the patient or follow-up care.

Doctors who wish to provide telephone or on-line services should consider carefully whether such a service will serve their patients' interests, and if necessary, seek advice from their professional association or medical defence society.

## Discussion and Recommendations

Most current professional codes and ethical guidelines for telemedicine explicitly discourage - sometimes even forbid - giving or offering any concrete medical advice via telecommunication and computer communication networks in the absence of a pre-existing patient-physician relationship. Against this background we have previously argued that: "Given the enormous patient demand for 'teleadvice' such restrictive guidelines should be reconsidered, as otherwise unqualified 'cyberquacks' offering dubious advice on a commercial basis [11] may take over. Thus, restrictive national provisions should perhaps be replaced by more liberal, less paternalistic international guidelines that do not prohibit any patient-physician interaction by e-mail but set international standards on proper teleadvice" [21].

As there is no clear-cut line between giving general information and practicing medicine on the Internet, ideally professional codes should not flatly forbid any teleadvice. Rather, physicians should have the responsibility to decide according to their ability and judgment on which point of the continuum the question is located, and how much and which information can be given to a patient given the constraints of the data available, the medium used, and the relationship established (Figure 3).

Based on the review of the literature and consultation with numerous experts, the following six principles for Type A teleadvice and teleconsultation are suggested.

### Six suggested principles for giving Type A teleadvice on the Internet

1. Physicians responding to patients' requests on the Internet should act within the limitations of telecommunication services and keep the global nature of the Internet in mind.
2. Not every aspect of medicine requires face-to-face communication or physical examination, thus teleadvice may be legitimate in some cases.
3. Requests for help, including unsolicited patient questions, should not be ignored, but dealt with in some manner.
4. Informed consent requires fair and honest labeling (disclaimers and disclosure).
5. Health professionals and information providers must maintain confidentiality.
6. Health professionals should define internal procedures and perform quality control measures.

#### Principle 1: Physicians responding to patients' requests on the Internet should act within the limitations of telecommunication services and keep the global nature of the Internet in mind.

As there is currently not enough evidence on the effects and effectiveness of teleadvice given to patients that contains information on diagnoses or treatment, physicians should not propose or attempt to diagnose or treat online.

##### Don'ts:

- Don't make a specific diagnosis. If you do mention possible diagnoses, always provide a disclaimer that this is only one of several possibilities, and that the final diagnosis can only be established by the treating physician.
- Don't prescribe medicines.
- Don't judge the appropriateness of therapeutic interventions or challenge the diagnosis given by other physicians without knowing the case in detail.
- Don't send out general information in the guise of individualized information. (For example, avoid "personalizing" general information by including the name of the sender automatically in the text.)
- Don't mention suspicions, especially those that could have severe consequences (e.g. possible diagnoses such as cancer). Keep the emotional impact of your advice in mind.
- Don't give detailed advice if you are not sure about the nationality or cultural background of the sender.

#### Principle 2: Not every aspect of medicine requires face-to-face communication.

While diagnosis and treatment should not be attempted online, there is much that online health professionals can legitimately do; for example, answering questions about the side effects of medicines and about whether certain symptoms need to be investigated.

Drug information is a good example. In several countries, for example at the United Kingdom's Trent Drug Information Centre, Leicester Royal Infirmary [22], there are already telephone help lines which provide information and advice on all aspects of drug treatment directly to the public via telephone. Such services may also be provided on the Internet.

Other questions that could be answered mainly fall under the field of preventive medicine such as lifestyle counseling, nutrition advice, primary injury and disease prevention, and questions regarding screening and health risk assessment including genetic counseling and tertiary prevention.

##### Things to do:

- Encourage patients to see a doctor if you feel the patient should, and if the patient seems to be reluctant for some irrational reason.
- Provide addresses of self-support groups and other organizations which may provide help and support.
- Provide addresses of specialists and hospitals.
- Answer general questions on side effects of medicines.
- Answer general questions on the compatibility of certain drugs and identify combinations of drugs which may pose problems.
- Give your opinion on whether certain symptoms need to be investigated.
- Answer questions on prevention of diseases and injuries.
- Recommend simple measures which may alleviate the problem.
- Try to identify questions the patient should ask himself to decide whether or not to see a doctor.
- Provide emotional support.
- Provide general information, e.g. disease fact sheets, the latest research results, and information on ongoing trials; but make clear that this is general information which may not apply to the patient's individual case and should be discussed with the treating physician.
- Refer to areas of uncertainties.

#### Principle 3: Requests for help, including unsolicited patient questions, should not be ignored, but dealt with in some appropriate manner.

Whether or not (and how) to react to an email largely depends in the content: "So how one deals with e-mail questions often depends on the content. Particularly inappropriate questions may be simply deleted, quickly disposed of without further thought" [23].

However, an interesting question is whether physicians have the ethical duty at least to try to help the patient to find more appropriate ways to answer his/her question. Currently, most physicians will actually simply delete the message without any attempts to help. In letter to the editor of the Archives of Dermatology a physician wrote in response to our call to establish guidelines [2]: "The appropriate resolution for the majority of unsolicited mail is the same as for unsolicited email: the wastebasket/delete button. Do not offer advice to someone you personally have not seen physically, touched and examined in real time" [24].

The letter writer brought forward the Hippocratic principle of "first do no harm" (primum nil nocere) to justify his position. However, we think that physicians have an ethical obligation not only to do no harm, but - if possible - to do good and to protect the patient, as has been pointed out in the following letter reply [25]:

While we are well aware of all the problems and pitfalls associated with giving advice under conditions of extremely limited information [11] and the problems of quality information on the Internet at large [26], we think that to react to these questions by simply discarding them is probably the worst of all possible alternatives. To delete them without having replied or even read the e-mail is not only disrespectful patients and rude, it also signifies an ignorance toward patients' concerns and is a slap in the face to those who argue that patients should be informed, educated, and encouraged to take responsibility for their own health. Already, patients are largely turning to the Internet because they think that physicians do not take enough time for their concerns [27].

Physicians (increasingly!) have an ethical responsibility to educate patients and consumers. To "respond" to patients' questions by deleting them seems much more unethical to us than giving a professional and courteous reply or forwarding the e-mail to a third party who can deal with the patient's concerns or questions. In any case, hitting the delete button is the opposite of what we would consider to act "for the good of my patients according to my ability and by judgment" (Hippocratic Oath).

Thus, physicians do have an ethical responsibility to read their email and to reply by helping the sender to find someone who can respond to their need. While this may not be always possible in practice, every effort should be made to minimize misunderstanding on the part of the patient, raising false hopes or causing potential harm by, for example, replying with a standard message saying that it is impossible to reply to every email.

The ethical duty to help may also be resolved by forwarding the email to an institution who is prepared to handle such requests (a "clearance center" for unsolicited emails has been proposed [21,25]). It is however essential that - if the email request is forwarded to a third party - the sender must give his or her consent. Alternately, the receiver may post on his website near his email address his policy of forwarding unsolicited patient emails to a third party.

Standard replies may for example contain URLs of useful and quality-assessed websites, addresses of self-support groups and professional organizations, or book titles and articles which contain further information (see Box 3).

**Example standard reply, used by HealthSCOUT (courtesy of Dr. Renner)**

Hello, ___________. Thank you for your recent request to HealthSCOUT. I receive many e-mails each week and will do my best to respond appropriately.

It's not right legally or ethically to practice medicine over the Internet. That may change in the future, but right now, the best advice you're going to get is from a face-to-face consultation with a physician.

Nevertheless, there are some distinct advantages to using the Internet for consumer health research, and HealthSCOUT is a very good place to do it.

If you do a key word search for ______, you'll probably find a number of HealthSCOUT items. In your case, for example, here is one of a number of articles found there____________. You can print out this and share it with your doctor.

Generally reliable Information about your question may be found elsewhere on the Internet on the following URL: www._____________.

Healthfinder___, Stars list___, medline___, others____.

If you want to really polish your skills at finding health information on the Internet look at www___________. Please make sure the information really applies to your specific condition by sharing it with your doctor.

If you are unable to find appropriate answers this way and you need further assistance, please call me at ________________.

If you get voice mail, leave your name, number and brief message, I will call you back.

Sincerely,

John H. Renner M.D. Chief Medical Officer, HealthSCOUT

Standard replies should be made clearly recognizable as standard replies. Under no circumstances should standard replies be made to appear to be individual replies.

Standard replies may be sent manually or automatically. If standard replies are sent manually, it should not take longer than 24 hours to respond.

If the patient gives a list of symptoms and asks for possible diagnoses, a standard reply could be sent, which points out that it is impossible to make a remote diagnosis without a complete medical history and examination. In some cases, standard replies are inappropriate and should be replaced by a more sensible personal email. A patient who received a standard reply (which contained the standard phrase to see a real doctor) replied angrily with the following email:

Dear Person, how insulting to have you tell me to seek a "real" doctor's advice. Obviously you did not read my e-mail. My sister has been given this diagnosis by a "real" doctor who told her she has to live with it!!!! I do not believe there is any condition one has "to live with", there is always help and hope in nature. That you choose not to offer any help or advice on where to find help is unfortunate. We merely want to find out more about this skin condition!

(Response from a patient to a standard reply of G.E.)

#### Principle 4: Informed consent requires fair and honest labeling.

Patients should at all times understand the nature and limitations of email consultations. In the context of "ask-the-doctor" services, terms and phrases labeling this service as "medical advice," or even "virtual hospital," may mislead (as they evoke certain associations leading to the impression that these services could replace a doctor visit) and should be avoided.

Disclaimers and full disclosure of all relevant information as specified below are crucial for informed consent and informed choice.

##### 4.1. Disclaimers and informed consent

Patients cannot always be expected to understand the nature and limitations of Internet consultations. Spielberg has pointed out that patient-initiated email does not automatically imply consent: "Simply because patients use email informally in other contexts does not mean that they understand the implications of communicating about sensitive medical topics" [4]. Thus, every effort should be taken to ensure that patients understand and explicitly consent to the implications of communicating with the physician, for example on issues such as:

- Storage and handling (in particular forwarding) of messages
- Security issues: Any sites that offer email addresses should contain a written statement regarding email security risks [8]

##### 4.2. Disclosure and informed choice

At a minimum, the following should be disclosed:

- The identities of those who will (have) read the patient's emails, and who will (have) answer(ed) them. If the physician delegates office staff to triage all incoming email, this should be disclosed on the website. If the physician uses a third-party service provider (e.g. a web site which handles back-end office tasks), that relationship should be made clear along with the potential for privacy and security violations related to third party.
- The qualifications of the responder
- Financial dependence / sponsoring
- Before the patient sends the request, he should be clear about all procedural aspects (e.g. are the emails forwarded, published, collected, etc.) and whether or not any costs will incur.

##### Principle 5: Health professionals and information providers must maintain confidentiality.

No medical interaction of any sort should generate the remotest possibility that an outside person or organization of any kind should be able to detect that the medical interaction had taken place.

If an "ask-the-doctor" service plans to publish users' questions, he or she should disclose this fact. Never should questions be published that could lead to identification of the sender without his or her explicit consent (in this case a general statement that questions may be published is not sufficient, but the individual must be contacted directly). Patients need to be informed about privacy issues and should know who reads his or her email if it is any person other than the doctor or addressee (see also Disclosure).

The physician should never communicate to a group of patients by email in such a way that all recipients are visible to one another (e.g. by using the carbon copy function [cc]).

##### Principle 6: Health professionals should define internal procedures and perform quality control measures.

Health professionals should define responsibilities within the institution and define procedures for triaging messages from unknown patients. Performing quality assessments assures that patients are receiving the correct and proper information.

### Conclusion

While the Internet offers huge opportunities for patients to educate themselves and to take responsibility for their own health, it also provides new challenges. One of these challenges is that patients and doctors alike still have to learn how to communicate with each other. Patients have to be educated that it is unethical to diagnose and treat over the Internet in the absence of a pre-existing patient-physician relationship, and if the interaction is limited to a single email. Likewise, physicians also sometimes break from the normal standards of care, and are tempted to make a diagnosis or even give therapeutic advice in the context of extremely limited information [1]. More research is needed to establish more evidence regarding situations in which teleadvice is beneficial and efficient.
